# Enhancement of Hydrogen Adsorption on Spray-Synthesized HKUST-1 via Lithium Doping and Defect Creation

**DOI:** 10.3390/ma16155416

**Published:** 2023-08-02

**Authors:** Masaru Kubo, Tomoki Matsumoto, Manabu Shimada

**Affiliations:** Department of Advanced Science and Engineering, Graduate School of Advanced Science and Engineering, Hiroshima University, Kagamiyama 1-4-1, Higashi-Hiroshima 739-8527, Hiroshima, Japansmd@hiroshima-u.ac.jp (M.S.)

**Keywords:** HKUST-1, metal–organic frameworks, hydrogen adsorption, lithium doping, defect creation

## Abstract

We prepared HKUST-1 (Cu_3_BTC_2_; BTC^3−^ = 1,3,5-benzenetricarboxylate) using a spray synthesis method with Li doping and defect created via partial replacement of H_3_BTC with isophthalic acid (IP) to enhance the H_2_ adsorption capacity. Li-doping was performed by incorporating LiNO_3_ in HKUST-1 via spray synthesis and subsequent thermal treatment for decomposing NO_3_^−^, which enhances H_2_ uptake at 77 K and 1 bar per unit mass and per unit area from 2.37 wt% and 4.16 molecules/nm^2^ for undoped HKUST-1 to 2.47 wt% and 4.33 molecules/nm^2^, respectively. Defect creation via the replacement of the BTC^3−^ linker with the IP^2−^ linker slightly in HKUST-1 skeleton did not affect H_2_ uptake. Both Li-doping and defect creation significantly enhanced H_2_ uptake to 3.03 wt%, which was caused by the coordination of Li ions with free carboxylic groups of the created defects via IP replacement.

## 1. Introduction

Global warming caused by CO_2_ emissions and the depletion of fossil fuels has created an urgent demand for environmentally friendly alternative energy systems [1]. Hydrogen (H_2_) is an important alternative energy source to fossil fuels. A H_2_-based energy system using fuel cells is expected to be central to a sustainable, recycling-oriented society owing to its high energy efficiency, low emissions of environmentally hazardous substances such as CO_2_ and NO_X_, and its ability to be reproduced using solar energy. However, the major challenge in realizing a H_2_ economy using fuel cells is its storage [2,3,4]. The U.S. Department of Energy requires on-board H_2_ storage systems of fuel-cell vehicles with 5.5 wt% or 40 kg/m^3^ of H_2_ tanks by 2025 [5].

Metal–organic frameworks (MOFs), which are crystalline porous materials consisting of coordination bonds between organic linkers and metal ions/clusters, have emerged as promising materials for H_2_ storage, owing to their exceptionally high surface area and pore volume [6,7,8]. At a low temperature (77 K) and high pressure, MOFs show exceptionally good H_2_ uptake, for example, the NU-1103 MOF has exhibited an H_2_ uptake of 13.0 wt% at 77 K and 100 bar [9]. However, because of the weak interaction between the MOF wall and H_2_ molecules, the H_2_ uptake at ambient temperatures is significantly smaller (just 2.8 wt% at 295 K and 100 bar for NU-1103 [9]). To overcome this weak interaction, the impregnation of strong adsorption sites into MOFs has been suggested as a way to improve the H_2_ uptake. Li^+^ ions are promising candidates for such an application owing to their low atomic weight and high affinity for molecular H_2_ as a result of charge-induced dipole interactions [10,11]. Theoretical investigations into Li-doped MOFs have suggested that H_2_ capacities of over 4.5 wt% under ambient conditions are achievable [12,13]. While experimental work is yet to reach those levels, the results thus far have been promising. For example, Li^+^-doped hydroxyl-MIL-53(Al) and MIL-53(Al) improved the H_2_ uptake at 77 K and 1 bar from 0.5 wt% to 1.7 wt% and from 1.66 wt% to 1.84 wt%, respectively [14,15]; Li^+^-exchanged NOTT-201 and MOF-5 exhibited the enhancement of H_2_ uptake from 0.96 wt% to 1.02 wt% and from 1.23 wt% to 1.39 wt%, respectively [16,17]; Li^+^-doped MIL-101 exhibited the enhancement of H_2_ uptake from 1.54 wt% to 2.65 wt% [18]. Methods for Li^+^ doping include the reduction of the MOF skeleton via organometallic lithium [14], Li exchange with protons of hydroxyl-modified MOFs [17,19] or anionic MOFs [16], and the thermal treatment of LiNO_3_-impregnated MOFs [15,18,20]. The thermal treatment of LiNO_3_-MOF, by which NO_3_^–^ anions in the LiNO_3_-impregnated MOF is thermal decomposed to NO and/or N_2_O gas at 200 °C [15], is preferred because Li doping can be achieved under moderate conditions compared with the other two methods.

Another way to enhance H_2_ uptake is to create defects in the MOF skeletons that lack coordination bonding between linkers and metals. The exposed linker and metals act as strong adsorption sites that enhance gas adsorption (H_2_ [21], CO [22], CO_2_ [23], and C_3_H_8_ [24]). By replacing the organic linker in the parent MOF structure with a partially fragmented linker, defects can be created without altering the MOF structure [25]. In another way, the number of defects can be changed via the synthesis procedure [26].

We recently developed a continuous spray synthesis method that forms MOF nanocrystals in a short time via evaporation of droplets of a sprayed MOF precursor solution [27,28]. Rapid MOF crystallization using this method forms many missing bonds that enhance the H_2_ uptake [26,29]. This method also enables the preparation of nanoparticle composite MOFs by adding nanoparticles to a sprayed solution [30,31]. Similar to nanoparticle incorporation, LiNO_3_ is also expected to be incorporated into HKUST-1 by adding it to the sprayed solution. Thus, the spray synthesis method enables both Li-doping and defect creation for the enhancement of the H_2_ storage capacity of MOFs.

In this study, we prepared a Li-doped HKUST-1 MOF (Cu_3_(BTC)_2_; H_3_BTC = 1,3,5-benzenetricarboxylic acid) via thermal decomposition of NO_3_^−^ in LiNO_3_-HKUST-1, synthesized via the spray-assisted synthesis method and subsequent thermal decomposition in a vacuum. In addition, we partially substituted H_3_BTC with isophthalic acid (IP = 1,3-benzenedicarboxylic acid) to form more defects in the HKUST-1 skeleton (defected-HKUST-1). Furthermore, we demonstrated both Li doping and defect creation into HKUST-1 via spray synthesis. We evaluated the crystallinity using X-ray diffraction (XRD) measurements, porous characteristics using nitrogen adsorption measurements, and H_2_ adsorption properties at 77 K.

## 2. Materials and Methods

### 2.1. Chemicals

Copper nitrate trihydrate (Cu(NO_3_)_2_·3H_2_O, 99%), lithium nitrate (LiNO_3_, 99%), 1 M HCl aqueous solution (for volumetric analysis), copper standard solution (Cu 1000), lithium standard solution (Li 1000), 20% deuterium chloride solution in D_2_O (DCl/D_2_O, 99.5%) N,N-dimethylformamide (DMF, 99%), and ethanol were purchased from Fuji-film Wako Pure Chemical Co. (Osaka, Japan), and 1,3,5-benzenetricarboxylate (H_3_BTC, 98%) and dimethyl sulfoxide-d_6_ (DMSO-d6, 99% contains 1% TMS) were purchased from Sigma-Aldrich (Burlington, MA, USA). All reagents were used without further purification.

### 2.2. Preparation of Precursor Solution of Li-Doped HKUST-1 (Li-HKUST-1)

The precursor solution for Li-HKUST-1 was a mixture of 5 mL of 0.75 mM Cu(NO_3_)_2_ aqueous solution and 10 mL of 0.25 mM H_3_BTC solution in a co-solvent containing 5 mL each of ethanol and N,N-dimethylformamide (DMF). First, 0.026–0.259 g of LiNO_3_ was added to the Cu(NO_3_)_2_ solution before mixing with the H_3_BTC solution. The Li/Cu ratios were 10, 20, and 200 mol%. Li-HKUST-1 samples obtained from these solutions are denoted as Li-10, Li-20, and Li-200, respectively. HKUST-1 without Li^+^ was synthesized using the same procedure but without the addition of LiNO_3_.

### 2.3. Preparation of Precursor Solutions of Defect-HKUST-1 (d-HKUST-1) and Li-Doped Defect-HKUST-1 (Li-d-HKUST-1)

An amount of isophthalic acid (IP) was added to the H_3_BTC solution. The ratio of IP/(IP+H_3_BTC) in the organic linker solution was varied as 6.25, 12.5, and 25 mol%, without changing the total organic linker concentration (0.25 mM); 10 mL of the organic solution was mixed with 5 mL of a 0.75 mM Cu(NO_3_)_2_ aqueous solution. The d-HKUST-1 samples obtained from these solutions are denoted as d6.25, d12.5, and d25, respectively. To prepare Li-d-HKUST-1, 0.026 g of LiNO_3_ was added to an aqueous Cu(NO_3_)_2_ solution at a 10 mol% of Li/Cu ratio. The Li-d-HKUST-1 samples obtained from these solutions are denoted as Li-d6.25, Li-d12.5, and Li-d25, respectively.

### 2.4. Spray Synthesis of Li-HKUST-1, d-HKUST-1, and Li-d-HKUST-1

Li-HKUST-1 and Li-d-HKUST-1 were synthesized using a homemade spray dryer, which consisted of a two-fluid nozzle, a spray chamber equipped with an inlet of swirling air to prevent deposition loss of droplets, a heating tube, and a filter holder [29]. The precursor solution was fed into the nozzle at a flow rate of 3 mL/min and sprayed with clean air at a flow rate of 12 L/min. Swirling air was supplied at a flow rate of 30 L/min and temperature of 180 °C. The sprayed droplets were then heated in a heating tube at 200 °C. The samples were then collected using a glass filter, washed with water and ethanol, ultrasonicated, and centrifugated to eliminate residual precursors [27]. After centrifugation, samples were dried at 60 °C for 3 h. Thermal decomposition of NO_3_^–^ ions was conducted at 200 °C under vacuum for 5 h.

### 2.5. Characterization

Powder XRD patterns were recorded on a Miniflex 600 instrument (Rigaku Corp., Tokyo, Japan) with Cu Kα radiation (wavelength 1.5406 Å). Inductively coupled plasma atomic emission spectrometry (ICP-AES) (SPS3000, Seiko Instrument Inc., Chiba, Japan) measurements were conducted for the quantitative analysis of the Li and Cu species in the samples. Before the ICP-AES measurements, 10 mg of each sample was dissolved in 1 mL of 1 M HCl aqueous solution. After dissolution, deionized water was carefully added to the solution until the volume reached 10 mL. The concentrations of Li and Cu in the sample solution were confirmed via the emission intensities at 670.784 and 327.396 nm for Li and Cu, respectively. Nitrogen adsorption–desorption measurements were carried out on an automated micropore gas analyzer (AUTOSORB-1-MP, Quantachrome Instruments, Boynton Beach, FL, USA) at 77 K after sample activation at 180 °C and 0.1 Pa for 6 h. The specific surface areas of the samples were calculated from nitrogen adsorption isotherms using the Brunauer–Emmett–Teller (BET) method in the range of 0.01 < *P*/*P*_0_ < 0.05. The micropore volumes were calculated using the *t*-plot method. To determine the IP ratio in the samples, the samples were digested via sonication in a mixture of DCl/D_2_O (35% solution) and DMSO-d_6_. The ^1^H NMR spectra were recorded on a Varian System 500 spectrometer. In the ^1^H NMR spectra, the ratio of the dicarboxylic acids was determined by comparing the integrals for the signal at δ = 8.64 ppm (H_3_BTC) with those for the signals at δ = 8.48, 8.18, and 7.69 ppm (IP).

## 3. Results

### 3.1. Li-HKUST-1

**Figure 1:**
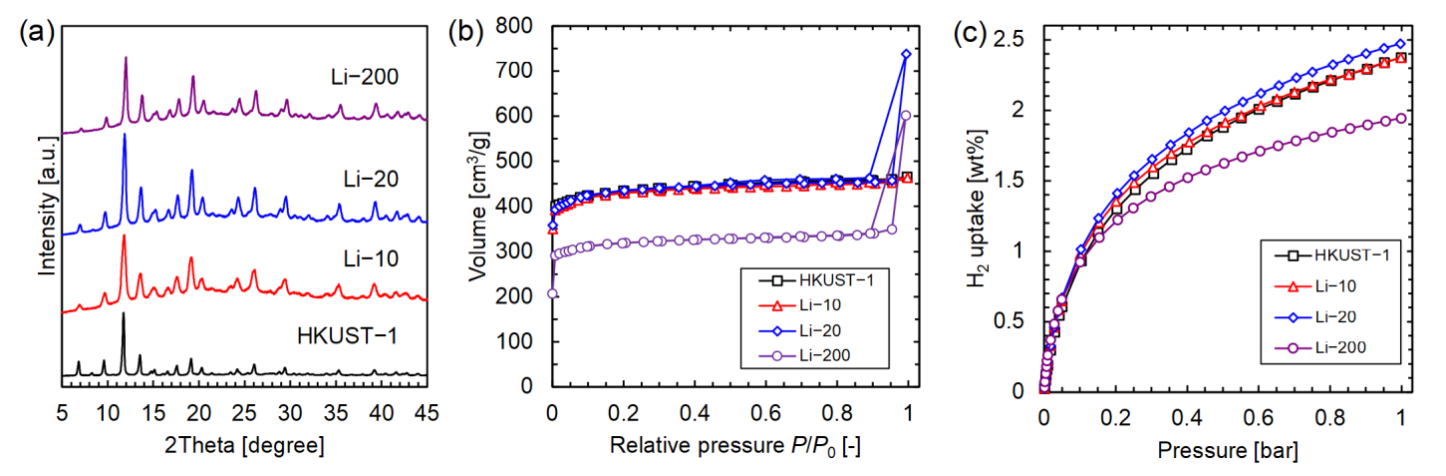
(**a**) XRD patterns, (**b**) N_2_ adsorption isotherms, and (**c**) H_2_ adsorption isotherms of HKUST-1 (black), Li-10 (red), Li-20 (blue), and Li-200 (purple).

Li-HKUST-1 was prepared by changing the Li/Cu ratio in the precursor solution to 10, 20, and 200 mol% (denoted as Li-10, Li-20, and Li-200, respectively). Figure 1a shows the XRD patterns of HKUST-1, Li-10, Li-20, and Li-200. All Li-HKUST-1 samples exhibits patterns attributed to HKUST-1, indicating that HKUST-1 is successfully formed even in the presence of LiNO_3_. The peak intensities of Li-10 and Li-20 are almost the same. However, that of Li-200 is slightly smaller, which indicates that the crystallinity of Li-200 is lower than that of Li-10 and Li-20; this is due to the excess amount of LiNO_3_ present. LiNO_3_ would be precipitated during droplet evaporation, which prevented the crystallization of HKUST-1 in droplets. Our previous study on the fabrication of the nanocomposites of HKUST-1 and Fe_3_O_4_ nanoparticles also showed that an excess amount of nanoparticles prevents crystallization [31].

Figure 1b shows the nitrogen adsorption isotherms of the samples. Li-10 and Li-20 show almost the same type-I isotherm as HKUST-1, whereas Li-200 shows a decrease in adsorption at all relative pressures. The BET surface areas, as summarized in Table 1, are 1705 m^2^/g for HKUST-1, 1548 m^2^/g for Li-10, 1706 m^2^/g for Li-20, and 1251 m^2^/g for Li-200. The small BET surface area of Li-200 is attributed to its low crystallinity, as revealed by the XRD pattern.

The Li/Cu ratios in Li-HKUST-1 were determined via elemental analysis using ICP-AES, and they were determined to be 0.29, 0.24, and 0.93 mol% in Li-10, Li-20, and Li-200, respectively, indicating the presence of Li in all three samples (Table 1). The Li/Cu ratio in Li-HKUST-1 is much lower than that in the precursor solution because of the dissolution and removal of LiNO_3_ by washing.

Figure 1c shows the H_2_ adsorption isotherms of the samples at 77 K. H_2_ uptakes of the samples are summarized in Table 1. The H_2_ uptake of HKUST-1 is 2.37 wt% at 1 bar, which is consistent with that of our previous result [29]. Li-10 exhibits the same H_2_ uptake as HKUST-1, even though its surface area was smaller. Li-20, the surface area of which is the same as that of HKUST-1, exhibits a higher H_2_ uptake of 2.47 wt%. Meanwhile, Li-200 exhibits a lower H_2_ uptake (1.95 wt%), owing to the decrease in surface area.

**Scheme 1:**
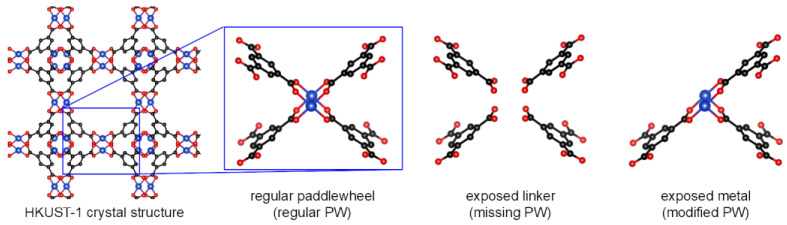
Different types of defects created via the spray synthesis method. (black, red, and blue balls represents hydrogen, oxygen, and copper atoms, respectively).

Previous studies on Li doping using LiNO_3_ suggested that Li ions were coordinated with the free carboxyl groups via solid-state NMR and XPS analyses [15,20]. Scheme 1 presents the possible defects generated via the spray synthesis method. The HKUST-1 crystal structure is constructed from paddlewheels formed via the coordination bonds of four BTC carboxyl groups and two Cu ions. Rapid crystallization during droplet evaporation would form two types of defects, which are exposed linkers and metals. Li ions would coordinate with free carboxyl groups of the exposed linker, which would act as the additional adsorption sites for H_2_ molecules.

### 3.2. Li-d-HKUST-1

To intentionally introduce defects into the HKUST-1 skeleton, the organic ligand H_3_BTC of HKUST-1 was partially replaced with IP. Defected-HKUST-1 (d-HKUST-1) was prepared by changing the ratio of IP/(IP+H_3_BTC) in the organic linker solution to 6.25, 12.5, and 25 mol% (denoted as d6.25, d12.5, and d25, respectively). Li-defected-HKUST-1 (Li-d-HKUST-1) was prepared with an IP/(IP+H_3_BTC) ratio and Li/Cu ratio of 10 mol% (denoted as Li-d6.25, Li-d12.5, and Li-d25, respectively). Table 2 summarizes the Li/Cu ratio, IP ratio, BET surface areas, and H_2_ uptakes at 77 K and 1 bar of the samples.

**Figure 2:**
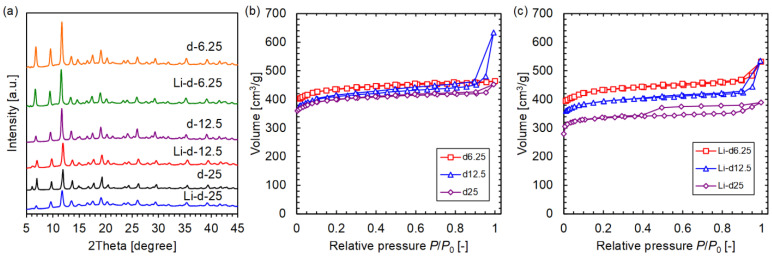
(**a**) XRD patterns and N_2_ adsorption isotherms for (**b**) d-HKUST-1 and (**c**) Li-d-HKUST-1.

Figure 2a shows the XRD patterns of d-HKUST-1 and Li-d-HKUST-1. All samples exhibit patterns attributed to HKUST-1, indicating that the replacement of H_3_BTC with IP and further Li impregnation does not affect the crystallization of HKUST-1 via the spray synthesis method. The Li/Cu ratio was analyzed using ICP-AES. It was found that the Li contents in Li-d6.25, Li-d12.5, and Li-d25 are 0.64, 0.85, and 0.51 mol%, respectively; these are higher than that in the case without IP (0.29 mol% of Li-10). The ratios of IP in d-HKUST-1 and Li-d-HKUST-1 were measured via ^1^H NMR after dissolution of the samples. The NMR spectra of d-HKUST-1 and Li-d-HKUST-1 are shown in Figures S1 and S2. The ratio of IP/(BTC+IP) calculated by integrating the signal at δ = 8.64 ppm for H_3_BTC with the signals at δ = 8.48, 8.18, and 7.69ppm for IP are listed in Table 2. Although the ratios are slightly lower than those in the precursor solution, IP was successfully introduced to the HKUST-1 crystal structure.

Figure 2b,c show the N_2_ adsorption isotherms of d-HKUST-1 and Li-d-HKUST-1, respectively. The BET surface areas of d6.25 and Li-d6.25 were 1714 and 1687 m^2^/g, respectively, which were similar to those of HKUST-1, indicating that there was no change in pore structure owing to low IP replacement. Conversely, with increasing IP ratio, the BET surface areas decreased to 1609, 1569, 1544, and 1330 m^2^/g for d12.5, d25, m^2^/g, Li-d12.5, and Li-d25, respectively. From the literature, the defect creation via IP replacement increased with BET surface area [25,32]. However, the results of this study show a lower BET surface area, which would be due to slightly lower crystallinity. The crystallization via the spray synthesis method occurred in <1 s. Presumably, the presence of IP, the incorporation of which is considered thermodynamically unstable, hindered crystallization in the short period and reduced the BET surface area. The lower IP ratio in the sample than that in the precursor solution is also due to the low crystallinity.

**Figure 3:**
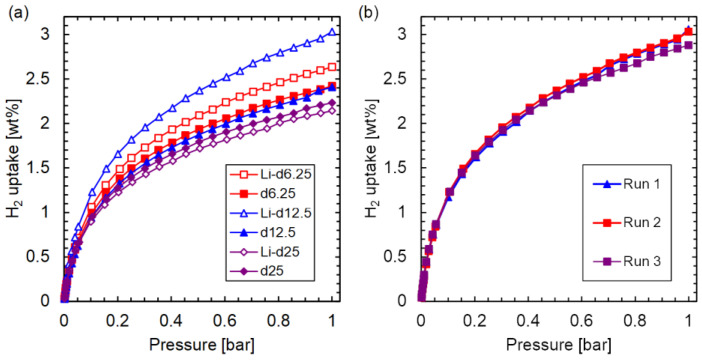
(**a**) H_2_ adsorption isotherms at 77 K of d-HKUST-1 and Li-d-HKUST-1 and (**b**) reproducibility test for Li-d12.5.

Figure 3a shows the H_2_ adsorption isotherms of d-HKUST-1 and Li-d-HKUST-1 at 77 K. The H_2_ uptake of d-HKUST-1 reaches 2.42 wt%, which is almost consistent with that of HKUST-1. Conversely, Li-d-HKUST-1 exhibits higher H_2_ uptake than HKUST-1 except for Li-d25. In particular, Li-d12.5 exhibits 3.03 wt% of H_2_ uptake at 77 K and 1 bar, which is 27% higher than that of HKUST-1. To confirm whether the improved H_2_ adsorption on Li-d12.5 is reproducible, the other two samples were prepared. Figure 3b shows the isotherms of the three samples. The H_2_ uptakes at 1 bar were 3.03, 3.06, and 2.88 wt% with no significant difference, indicating the reproducibility of the enhancement of H_2_ adsorption by combining Li-doping and defect creation via IP replacement.

**Scheme 2:**
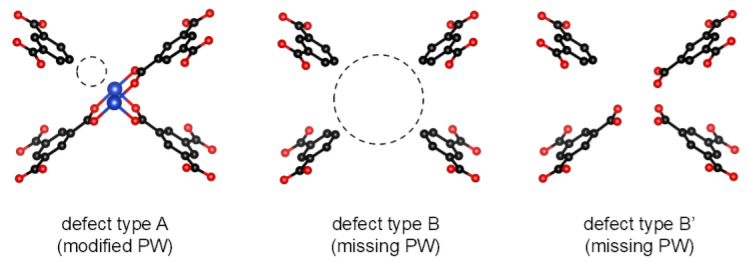
Possible defects in d-HKUST-1. (black, red, and blue balls represents hydrogen, oxygen, and copper atoms, respectively).

The enhancement of H_2_ uptake in Li-d-HKUST-1 is discussed by considering defects. The possible defects in d-HKUST-1 and Li-d-HKUST-1 are the three additional defects shown in Scheme 2, as well as the two defects shown in Scheme 1. Defect types A and B have been proposed in the literature [22,25,32]. Using Cu(NO_3_)_2_ as the copper source, type B defects are more abundant [25]. However, considering the amount of IP, it is unlikely that all four linkers were IP. There may be defects, such as type B’, with exposed BTC linkers. Similar to the discussion on Li-HKUST-1, the free carboxylic groups of type B’ defects coordinated with that of Li ions, which act as additional adsorption sites on H_2_ molecules, resulting in an increase in H_2_ uptake.

Finally, we compared the results of other studies on Li doping into MOF. Table S1 summarizes the Li-doping agents, the ratio of Li to metal, BET surface area, and H_2_ uptakes of other Li-doped MOFs. Compared with the improvements of 10–72% in H_2_ uptake by Li-doping, an improvement of 27% for Li-d12.5 is not significantly high. However, the absolute H_2_ uptake of Li-d12.5 is 3.03 wt% at 77 K and 1 bar, and this is higher than those of most of the Li-doped MOFs. Furthermore, the amount of Li is lower than those of other Li-doped MOFs, and therefore, there is potential for further improvements in H_2_ uptake by increasing the Li-doping amounts.

## 4. Conclusions

Li doping and defect creation were performed on spray-synthesized HKUST-1 to improve its H_2_ adsorption properties. Li-doping was conducted via LiNO_3_ incorporation and subsequent thermal decomposition of NO_3_^−^. Li-HKUST-1 resulted in the enhancement of H_2_ uptake from 2.37 wt% for the non-treated MOF to 2.47 wt%, at 77 K and 1 bar. Defect creation in HKUST-1 was conducted via partial replacement of the original linker, H_3_BTC, with a fragmented linker, IP, which resulted in a H_2_ uptake up to 2.42 wt%. Combining Li-doping and defect creation significantly improved H_2_ uptake to 3.03 wt% (27% enhancement). This research provides a facile and efficient process for the fabrication of a MOF-based H_2_ storage material with additional adsorption sites resulting from the presence of Li cations and defects.

## Figures and Tables

**Table 1:** Summary of the elemental analysis, and N_2_ and H_2_ adsorption measurements for Li-HKUST-1.

Sample	Li/Cu (mol%)	BET Surface Area (m^2^/g)	H_2_ Uptake at 77 K and 1 Bar (wt%)
HKUST-1	0	1705	2.37
Li-10	0.29	1548	2.37
Li-20	0.24	1706	2.47
Li-200	0.93	1251	1.94

**Table 2:** Summary of elemental analysis, and N_2_ and H_2_ adsorption measurements for d-HKUST-1 and Li-d-HKUST-1.

Sample	Li/Cu (mol%)	IP/(BTC+IP) (%)	BET Surface Area (m^2^/g)	H_2_ Uptake at 77 K and 1 Bar (wt%)
d6.25	0	4.8	1714	2.42
d12.5	0	10.3	1609	2.41
d25	0	22.2	1569	2.23
Li-d6.25	0.64	4.2	1687	2.63
Li-d12.5	0.85	10.9	1544	3.03
Li-d25	0.51	19.9	1330	2.14

## Data Availability

Data are contained within the article.

## Supplementary Materials

The following supporting information can be downloaded at: https://www.mdpi.com/article/10.3390/ma16155416/s1, References [33,34,35] are cited in the supplementary materials; Figures S1 and S2: ^1^H NMR spectra of the digested samples; Table S1: Comparison of Li doping agent, Li/metal ratio, surface area, and H_2_ uptake at 77 K and 1 bar for other Li-doped MOFs.
