# Supplementary material for: Enhancement of Hydrogen Adsorption on Spray-Synthesized HKUST-1 via Lithium Doping and Defect Creation
Source: Materials (Basel). 2023 Aug 2;16(15):5416. doi: 10.3390/ma16155416 (PMC10419904; doi:10.3390/ma16155416)
Supplement: Supplementary file 1 [file materials-16-05416-s001.zip › materials-2518194-supplementary.pdf]

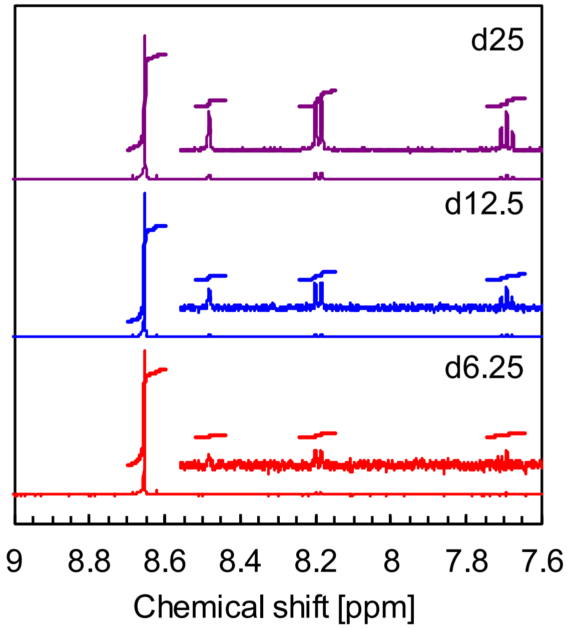

Figure S1 ^{1}H NMR spectra of d-HKUST-1 digested in DCl/DMSO-d6 mixture.

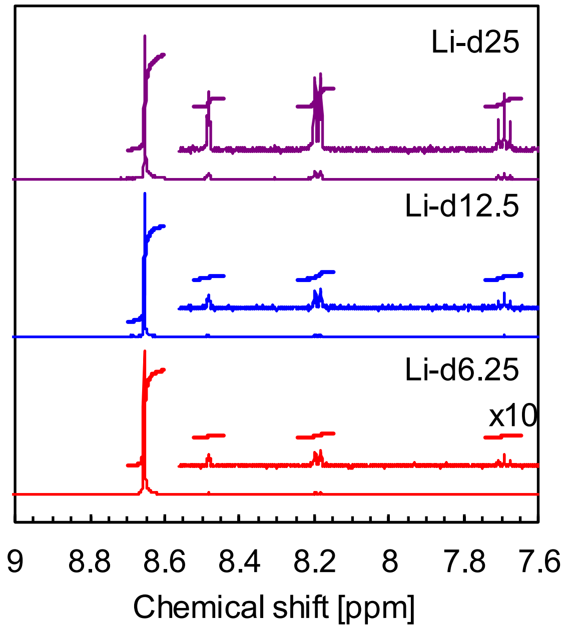

Figure S2 ^{1}H NMR spectra of Li-d-HKUST-1 digested in DCl/DMSO-d6 mixture.

Table S1 Comparison of Li doping agent, Li/metal ratio, surface area, and H_2 uptake at 77 K and 1 bar for other Li-doped MOFs.

MOFs	Li agent	Li/metal [mol%]	BET surface area [m²/g]	H_2 uptake [wt%]	Ref.
HKUST-1	0	0	1705	2.37	This work
Li-10	$Li(NO_3)_2$	0.29	1548	2.37	This work
Li-20	$Li(NO_3)_2$	0.24	1706	2.47	This work
Li-200	$Li(NO_3)_2$	0.93	1251	1.94	This work
Li-d6.25	$Li(NO_3)_2$	0.64	1687	2.63	This work
Li-d12.5	$Li(NO_3)_2$	0.85	1544	3.03	This work
Li-d25	$Li(NO_3)_2$	0.51	1330	2.14	This work
$Me_2NH_2[In(L_1)]^a$	–	0	820	1.39	[33]
$Li_{0.5}(H_3O)_{0.5}[In(L_1)]^a$	LiCl	50	1024	1.66	[33]
$[H_2ppz][In_2(L_2)_2]^b$	–		180	0.96	[16]
$Li_{1.5}[H_3O]_{0.5}[In_2(L_2)_2]^b$	LiCl	75	580	1.02	[16]
$Zn_2(NDC)_2$(diPyTz)	–	0	400	1.12	[34]
Li · $Zn_2(NDC)_2$(diPyTz)	Li-Naph[c]	5	526	1.46	[34]
DO-MOF		0	810	1.23	[19]
DO-MOF-Li0.20	LiO*t*Bu[d]	10	840	1.32	[19]
DO-MOF-Li2.62	LiO*t*Bu[d]	262	270	0.77	[19]
HKUST-1	–	0	1587	2.41	[35]
Li-HKUST-1	Li-Naph[c]	7	795	3.50	[35]
MIL-101(Cr)	–	0	2537	2.37	[35]
Li-MIL-101(Cr)	Li-Naph[c]	1	1840	3.39	[35]
MIL-53(Al)	–		976	1.66	[15]
Li-MIL-53(Al)	$Li(NO_3)_2$	4	958	1.84	[15]
MOF-5-OH-0.38	–	0	3220	1.23	[17]
MOF-5-Oli	LDA[e]	19	3190	1.39	[17]
RHA-MIL-101	-	0	2525	1.54	[18]
0.06Li-RHA-MIL-101	$Li(NO_3)_2$	0.06 wt%	2942	2.65	[18]
0.3Li-RHA-MIL-101	$Li(NO_3)_2$	0.32 wt%	2141	2.42	[18]
0.6Li-RHA-MIL-101	$Li(NO_3)_2$	0.63 wt%	1825	1.69	[18]
6.0Li-RHA-MIL-101	$Li(NO_3)_2$	6.09 wt%	1073	1.58	[18]

[a]H_4L_1 = biphenyl-3,3′,5,5′-tetracarboxylic acid; [b]H_4L_2 = 1,1′,4′,1″,4″,1‴-quaterphenyl-3,5,3‴,5‴-tetracarboxylic acid; [c]Li-Naph = lithium naphthalene; [d]LiOtBu = Lithium tert-Butoxide; [e]LDA = Lithium diisopropylamide

References

15. Kubo, M.; Shimojima, A.; Okubo, T. Effect of Lithium Doping into MIL-53(Al) through Thermal Decomposition of Anion Species on Hydrogen Adsorption. *J. Phys. Chem. C* **2012**, *116*, 10260–10265.

16. Yang, S.; Lin, X.; Blake, A.J.; Walker, G.S.; Hubberstey, P.; Champness, N.R.; Schröder, M. Cation-Induced Kinetic Trapping and Enhanced Hydrogen Adsorption in a Modulated Anionic Metal–Organic Framework. *Nat. Chem.* **2009**, *1*, 487–493.
17. Kubo, M.; Hagi, H.; Shimojima, A.; Okubo, T. Facile Synthesis of Hydroxy-Modified MOF-5 for Improving the Adsorption Capacity of Hydrogen by Lithium Doping. *Chem. Asian J.* **2013**, *8*, 2801–2806.
18. Panchariya, D.K.; Kumar, E.A.; Singh, S.K. Lithium-Doped Silica-Rich MIL-101(Cr) for Enhanced Hydrogen Uptake. *Chem. Asian J.* **2019**, *14*, 3728–3735.
19. Mulfort, K.L.; Farha, O.K.; Stern, C.L.; Sarjeant, A.A.; Hupp, J.T. Post-Synthesis Alkoxide Formation within Metal-Organic Framework Materials: A Strategy for Incorporating Highly Coordinatively Unsaturated Metal Ions. *J. Am. Chem. Soc.* **2009**, *131*, 3866–3868.
33. Yang, S.; Lin, X.; Blake, A.J.; Thomas, K.M.; Hubberstey, P.; Champness, N.R.; Schröder, M. Enhancement of H_2 Adsorption in Li^+-Exchanged Co-Ordination Framework Materials. *Chem. Commun.* **2008**, 6108–6110.
34. Mulfort, K.L.; Wilson, T.M.; Wasielewski, M.R.; Hupp, J.T. Framework Reduction and Alkali-Metal Doping of a Triply Catenating Metal-Organic Framework Enhances and Then Diminishes H_2 Uptake. *Langmuir* **2009**, *25*, 503–508.
35. Xiang, Z.; Hu, Z.; Yang, W.; Cao, D. Lithium Doping on Metal-Organic Frameworks for Enhancing H_2 Storage. *Int. J. Hydrogen Energy* **2012**, *37*, 946–950.
